# Tailored Fermentation of Large Yellow Croaker Surimi Balls with Direct Vat Set Starters: Effects on Physicochemical and Sensory Properties

**DOI:** 10.3390/foods14223825

**Published:** 2025-11-08

**Authors:** Shumin Liu, Yijia Deng, Shengjun Chen, Ruosong Yao, Shuangping Li, Peiyi Ye, Rundong Wang, Ahmed S. M. Saleh, Jianrong Li

**Affiliations:** 1 College of Food Science and Engineering, Lingnan Normal University, Zhanjiang 524048, China; liushuminlsm@163.com (S.L.); 13124933050@163.com (R.Y.); alive917@163.com (S.L.); yepeiyi2003@163.com (P.Y.); ahmed.saleh@aun.edu.eg (A.S.M.S.); 2 National & Local Joint Engineering Research Center of Storage, Processing and Safety Control Technology for Fresh Agricultural and Aquatic Products (Sub-Center), Zhanjiang 524048, China; ljr6491@163.com; 3 Key Laboratory of Aquatic Product Processing, Ministry of Agriculture and Rural Affairs, South China Sea Fisheries Research Institute, Chinese Academy of Fishery Sciences, Guangzhou 510300, China; 4 College of Food Science and Engineering, Bohai University, Jinzhou 121013, China

**Keywords:** fermented surimi, starter culture, texture improvement, flavor enhancement, protein stability

## Abstract

This study investigated the effects of direct vat set commercial yoghurt starter (B) and yeast starter (Y) on the quality of fermented large yellow croaker surimi balls, with natural fermentation (CTR) as a control. Surimi products were inoculated and fermented at 25 °C for 4 h, then analyzed for physicochemical, sensory, and oxidative properties. Yoghurt starter significantly inhibited protein oxidation, as indicated by the highest sulfhydryl content (9.10 nmol/mg protein, *p* < 0.05), improved textural properties (hardness was 28% higher than CTR, *p* < 0.05), and promoted a balanced flavor profile, accompanied by the highest equivalent umami concentration (1.66%, *p* < 0.05). However, B also caused the greatest MDA accumulation (1.49 mg/kg, *p* < 0.05), reflecting enhanced lipid oxidation. By comparison, Y enhanced umami primarily through significant enrichment of aspartic acid (53.88 mg/100 g, *p* < 0.05) and accelerated nucleotide degradation, resulting in the highest AMP and hypoxanthine levels (*p* < 0.05). These advantages were offset by severe protein carbonylation (54.32 nmol/mg protein, *p* < 0.05) and evident color deterioration. Sensory analysis revealed no significant difference between B and CTR (*p* > 0.05), whereas Y received significantly lower acceptance scores (*p* < 0.05) due to impaired color and taste. These findings suggest that B is a promising starter for improving texture and flavor in fermented surimi balls, while Y, despite enhancing umami and controlling lipid oxidation, negatively affects color, texture, and protein stability.

## 1. Introduction

*Larimichthys crocea*, commonly known as the large yellow croaker, is a marine species belonging to the family *Sciaenidae* and genus *Larimichthys*. It is one of the most economically important fish species in East Asia and currently ranks as the most extensively farmed seawater fish in China, as well as one of the eight major exported aquaculture products [1,2]. In addition to its tender texture, croaker is rich in high-quality protein, polyunsaturated fatty acids (such as DHA and EPA), minerals, and vitamins, conferring both desirable sensory qualities and notable dietary and health benefits [3,4]. Despite these attributes, the product portfolio of croaker remains limited. Domestic markets are largely supplied with fresh-ice products, while exports rely primarily on three conventional forms: fresh, frozen, and salt-cured products [5]. The lack of product diversification, low levels of value-added processing, severe product homogenization, and insufficient fundamental research constitute critical bottlenecks restricting the high-value utilization of croaker [1,5]. Addressing these challenges requires technological innovation and industrial upgrading.

Surimi-based products represent a promising direction for the value-added processing of croaker, although they have not yet been extensively studied or commercially exploited [5]. Surimi products are rich in high-quality complete protein, low in fat, and high in unsaturated fatty acids, aligning well with modern health-oriented consumption trends and presenting strong market potential [6,7]. However, croaker surimi often exhibits weak gelation properties and pronounced fishy odors. Recently, microbial fermentation technology has been introduced into surimi processing to address these issues. Inoculation with starter cultures has been shown to effectively improve gel texture and flavor profiles [7]. The fermentation process not only promotes the breakdown of proteins into small peptides and free amino acids, enhancing umami and reducing bitterness, but also increases protein digestibility and imparts unique aromatic characteristics [8,9]. Currently, microorganisms commonly used in meat fermentation include lactic acid bacteria (LAB), coagulase-negative staphylococci, yeast, and molds [10,11]. Among these, LAB are the most widely applied in surimi fermentation due to their ability to acidify the matrix, inhibit spoilage bacteria, reduce off-odors, and enhance overall flavor. For example, Riebroy et al. [12] demonstrated that LAB significantly increased the gel strength of surimi from yellowfin sea bream. Similarly, Li et al. [13,14] reported that co-culture fermentation with *Latilactobacillus sakei* and *Pediococcus acidilactici* effectively suppressed the formation of biogenic amines in tilapia surimi, while simultaneously increasing the content of umami amino acids and improving the volatile flavor composition.

Although microbial fermentation technology has been preliminarily applied in surimi processing, research specifically focused on croaker surimi remains limited. Moreover, conventional starter cultures usually require complex activation and propagation procedures, which demand stringent production conditions and operational expertise, thereby restricting their industrial-scale application. In contrast, direct vat set (DVS) commercial cultures offer several advantages, including high safety, elevated viable cell counts, extended shelf life, and excellent strain stability. Their utilization in food fermentation eliminates the need for tedious pre-cultivation steps, ensures consistent fermentation performance, and significantly enhances production efficiency along with improved flavor and sensory uniformity [15,16,17]. Currently, DVS cultures have been widely adopted in various sectors such as dairy products [18], baked goods [19], fermented sausages [15], wine making [20], and pickled vegetables [21]. However, the application of non-aquatic originated DVS starters (e.g., yoghurt cultures and yeast strains from dairy or brewing backgrounds) in croaker surimi fermentation remains largely unexplored. The metabolic behaviors of these exogenous microorganisms within the surimi matrix, their overall impact on product quality, and particularly systematic evaluations based on multi-dimensional quality attributes remain insufficiently studied.

We hypothesized that non-aquatic DVS starters (yoghurt and yeast) would differentially modulate the texture, flavor, and oxidative stability of large yellow croaker surimi via distinct metabolic pathways. Therefore, this study aimed to systematically compare their effects through a comprehensive profiling of sensory, physicochemical, and oxidative properties, and to provide a theoretical basis for the value-added utilization of *L. crocea*.

## 2. Materials and Methods

### 2.1. Materials and Reagents

Iced fresh large yellow croaker (*Larimichthys crocea*) was procured from a local aquatic products market (Zhanjiang, China). A direct vat set (DVS) commercial yoghurt starter culture, containing *Lactobacillus delbrueckii* subsp. *Bulgaricus* and *Streptococcus salivarius* subsp. *thermophilus*, was supplied by Wecare-Probiotics Co., Ltd. (Suzhou, China). Active dry yeast (*Hansenula* spp.) for flavor enhancement was obtained from Angel Yeast Co., Ltd. (Yichang, China). Food-grade non-iodized refined salt was sourced from Hunan Xuetian Salt Technology Development Co., Ltd. (Changsha, China). Sugar and monosodium glutamate were purchased from Guangdong Fuzheng Donghai Food Co., Ltd. (Guangzhou, China) and Guangzhou Aosang Monosodium Glutamate Food Co., Ltd. (Guangzhou, China), respectively. Fresh eggs and ginger were acquired from a local supermarket (Yanling Supermarket, Zhanjiang, China). All ingredients used in the study were of food grade.

Reference standards for flavor nucleotides and free amino acids were obtained from the National Research Center (Beijing, China). HPLC-grade methanol and acetonitrile were purchased from Thermo Fisher Scientific, Inc. (Shanghai, China). An enhanced BCA protein assay kit (P0010S) was procured from Beyotime Biotechnology (Shanghai, China). Ultra-pure water was produced in-house using a UV-TOC water purification system (Thermo Fisher Scientific, Inc., Carlsbad, CA, USA). All other chemicals used were of analytical grade.

### 2.2. Preparation of Fermented Large Yellow Croaker Surimi Balls

Fresh iced large yellow croaker (500–750 g per fish) was decapitated, descaled, and eviscerated. The flesh was manually separated from the skin starting from the head to the tail. The skinned fillets were rinsed two to three times with clean water, drained, and sliced. The slices were then ground using a meat grinder. Based on the raw fish mass, the following ingredients were added: 30% egg white, 3% salt, 3% sugar, 2% monosodium glutamate (MSG), and 50% frozen ginger juice (prepared at a ginger-to-water ratio of 1:10, *w*/*v*). The mixture was thoroughly homogenized by chopping.

The surimi was divided into three groups: two inoculated groups and one control. For the inoculated groups, 3% (*w*/*w*) of either yoghurt starter (B) or dry yeast (Y) was added separately. The control group (CTR) received no starter culture. Each mixture was mixed uniformly, transferred into sterilized containers, sealed, and fermented at 25 °C for 4 h in a constant temperature incubator. After fermentation, the surimi was shaped into spherical balls approximately 3 cm in diameter. The balls were placed in cold water, heated over medium-high heat until the water reached 100 °C, then simmered over low heat for 2–3 min. Once the surimi balls floated completely to the surface, they were immediately removed and transferred into cold water to cool rapidly to room temperature. After draining, the surimi balls were divided into three portions: one for sensory evaluation, one stored at 4 °C for texture and color measurement, and the remainder ground again with a meat grinder, packaged in food-grade bags, and stored at −20 °C for further analysis.

### 2.3. Determination of pH and Moisture Content

The pH was determined according to the method described by Liu et al. [8] with slight modifications. Briefly, 2 g of surimi balls was accurately weighed, homogenized with 18 mL of distilled water at 10,000× *g* for 1 min, and then centrifuged at 1000× *g* for 10 min. The pH of the resulting supernatant was measured using a calibrated pH meter (PHS-3C, Inesa Scientific Instrument Co., Ltd., Shanghai, China). The moisture content was determined by the direct drying method. Specifically, 2 g of surimi balls was accurately weighed and dried at 105 °C to constant weight according to the AOAC method [22].

### 2.4. Color Measurement

Color parameters were measured according to the method described by Patinho et al. [23] using a chroma meter (CR-400, Konica Minolta Investment Ltd., Shanghai, China) with a D65 light source. The instrument was calibrated with a standard white tile prior to measurement. Samples were cut into uniform cubes (2 cm × 2 cm × 2 cm) for analysis. The CIE color values, including lightness (L*), redness (a*), and yellowness (b*), were recorded. Nine replicates were measured for each sample, and mean values were calculated. The whiteness (W) of surimi balls was calculated using the following Formula (1):

(1) $$W=100-\sqrt{{\left(100-L^{*}\right)}^{2}+a^{*2}+b^{*2}}$$

### 2.5. Texture Profile Analysis (TPA)

Texture properties of the surimi balls were evaluated using a texture analyzer (TA-XT, Stable Micro Systems Ltd., Godalming, UK) in Texture Profile Analysis (TPA) mode according to the method of Xiong et al. [24]. The measurements were performed using a 50-mm diameter cylindrical probe (P/50) with a test speed of 1.0 mm/s, 50% deformation, and a trigger force of 5 g. Six replicates were performed for each sample.

### 2.6. Sensory Evaluation

Sensory evaluation was performed based on the method described by Dai et al. [25] with minor modifications. A trained panel comprising 30 members (15 males and 15 females, aged 20–30 years) from the Marine Food Processing and Utilization Research Team participated in the analysis. Prior to evaluation, samples were coded with random three-digit numbers and cooled to room temperature. Panelists evaluated the samples based on color, appearance, texture, flavor, and taste. Evaluation criteria are summarized in Table A1.

### 2.7. Determination of Free Amino Acid Content

Free amino acids were extracted and derivatized according to previously described procedures [26,27]. Briefly, 2.0 g of sample was homogenized with 20 mL of phosphate buffer (pH 7.0) to extract free amino acids. The homogenate was centrifuged (1000× *g*, 10 min), and the supernatant was collected. The supernatant was then filtered through a 0.45 μm membrane filter. For derivatization, 100 μL of the filtrate was sequentially mixed with 100 μL of triethylamine-acetonitrile solution and 100 μL of phenyl isothiocyanate-acetonitrile solution. The mixture was incubated at room temperature for 1 h, followed by the addition of 300 μL n-hexane. After centrifugation, 100 μL of the lower aqueous phase was diluted with 900 μL ultrapure water and analyzed using an HPLC system (LC-20AD, Shimadzu Corporation, Kyoto, Japan) equipped with a Venusil AA column (4.6 mm × 250 mm, 5 μm). The column temperature was maintained at 40 °C, and the detection was carried out at 254 nm. The mobile phase consisted of sodium acetate-acetonitrile solution (A), and the mobile phase (B) was 80% acetonitrile in water. The flow rate was 1.0 mL/min, and the injection volume was 10 μL. All analyses were performed in triplicate.

### 2.8. Determination of Flavor Nucleotide Content

Nucleotides were extracted based on a previously described method [28,29]. Briefly, 4 g of sample was homogenized with 20 mL of pre-cooled 10% perchloric acid (PCA) at 10,000× *g* for 1 min, followed by centrifugation at 8000 r/min and 4 °C for 10 min. The residue was re-extracted with 10 mL of 5% PCA. The combined supernatant was neutralized to pH 5.8 using 10, 1, and 0.1 mol/L NaOH solutions, diluted to 50 mL with ultra-pure water, and filtered through a 0.22 μm membrane. Analysis was performed using an HPLC system equipped with a Shimadzu ODS-3 C18 column (4.6 mm × 250 mm, 5 μm). The column temperature was 30 °C, the detection wavelength was 254 nm, and the flow rate was 1.0 mL/min. Mobile phase A consisted of 20 mmol/L KH_2_PO_4_/K_2_HPO_4_ buffer (1:1, *v*/*v*, pH 5.8), and mobile phase B was 100% methanol. All samples were analyzed in triplicate.

### 2.9. Umami Assessment

The taste activity value (TAV) and equivalent umami concentration (EUC) were calculated based on the contents of free amino acids and flavor nucleotides obtained in Section 2.7 and Section 2.8, using Formulas (2) and (3), respectively [28]:

(2) $$TAV=\frac{C}{T}$$

(3) $$EUC=\sum a_{i}b_{i}+1218(\sum a_{i}b_{i})(\sum a_{j}b_{j})$$

where *C* is the content of taste compound (mg/100 g); *T* is the threshold of taste compound (mg/100 g); *EUC* is the concentration of monosodium glutamate (MSG); *a_i_* is the concentration of each umami amino acid (Asp or Glu, g/100 g); *a_j_* is the concentration of each umami flavor nucleotide (IMP and AMP, g/100 g); *b_i_* is the relative concentration (RUC) of each umami amino acid to MSG (Glu is 1, Asp is 0.077); *b_j_* is the RUC of each umami flavor nucleotide compared to IMP (IMP is 1, AMP is 0.18); 1218 is the synergistic constant.

### 2.10. Determination of Malondialdehyde (MDA), Carbonyl and Sulfhydryl Content

MDA content was determined using the HPLC method specified in GB 5009.181-2016 [30]. Briefly, 5 g of sample was homogenized with 50 mL of trichloroacetic acid solution, incubated at 50 °C for 30 min, filtered, and reacted with thiobarbituric acid (TBA) solution. The reaction mixture was heated at 90 °C for 30 min, cooled, filtered, and subjected to HPLC analysis under the following conditions: Shimadzu ODS-3 C18 column (4.6 mm × 250 mm, 5 μm), column temperature, 30 °C; detection wavelength, 532 nm; mobile phase, 0.01 mol/L ammonium acetate:methanol (70:30, *v*/*v*); flow rate, 1.0 mL/min; injection volume, 10 μL. MDA content was calculated according to Equation (4):

(4) $$\mathrm{MDA}\;\mathrm{content}\;(\mathrm{mg}/\mathrm{kg})\;=\;\frac{C\times V\times 1000}{m\times 1000}$$

where *C* is the concentration from the standard curve (μg/mL), *V* is the volume of the sample solution (mL), and *m* is the sample mass (g).

Carbonyl content was determined following the method of Wang et al. [31] with minor modifications. The sample homogenate was reacted with 2,4-dinitrophenylhydrazine (DNPH), and absorbance was measured spectrophotometrically at 370 nm. Carbonyl content was calculated using Equation (5):

(5) $$\mathrm{Carbonyl}\;\mathrm{content}\;(\mathrm{nmol}/\mathrm{mg}\;\mathrm{protein})\;=\;\frac{A}{22000\times C\times D}\times 10^{6}$$

where *A* is the absorbance, *C* is the protein concentration (mg/L), *D* is the dilution factor, and 22,000 is the molar extinction coefficient (L·mol^−1^·cm^−1^).

Sulfhydryl content was determined following the methods of Zhang et al. [32] and Cui et al. [33] with modifications. The sample was homogenized, reacted with 5,5′-dithiobis-(2-nitrobenzoic acid) (DTNB), and absorbance was measured at 412 nm. Sulfhydryl content was calculated using Equation (6):

(6) $$\mathrm{Sulfhydryl}\;\mathrm{content}\;(\mathrm{nmol}/\mathrm{mg}\;\mathrm{protein})\;=\;\frac{A\times D}{13600\times C}\times 10^{6}$$

where *A* is the absorbance, *C* is the protein concentration (mg/L), *D* is the dilution factor, and 13,600 is the molar extinction coefficient (L·mol^−1^·cm^−1^).

### 2.11. Determination of Sodium (Na^+^) and Potassium (K^+^)

Na^+^ and K^+^ contents were determined using flame atomic absorption spectrometry according to GB 5009.91-2017 [34]. Briefly, approximately 0.5 g of sample was digested with nitric acid in a microwave digestion system (MARS6, CEM Corp., Matthews, NC, USA). After digestion, the solution was evaporated to remove excess acids, diluted to 50 mL with deionized water, and analyzed using an atomic absorption spectrometer (ZEEnit 700p, Analytik Jena AG, Jena, Germany) at wavelengths of 589.0 nm (Na^+^) and 766.5 nm (K^+^). The Na^+^ and K^+^ contents were calculated using Equation (7):

(7) $${\mathrm{Na}}^{+}/K^{+}\;\mathrm{content}\;(\mathrm{mg}/100\;g)\;=\;\frac{\left(\rho -\rho_{0}\right)\times V\times f\times \;100}{m\times 1000}$$

where *ρ* is the element concentration in the sample (mg/L), *ρ*_0_ is the element concentration in the blank (mg/L), *V* is the volume of the sample solution (mL), *F* is the dilution factor, and *m* is the sample mass (g).

### 2.12. Statistical Analysis

All experiments were performed in triplicate (at least three independent replicates). Data are expressed as mean ± standard deviation (SD) and were plotted using Origin (version 2024, OriginLab Corp., Northampton, MA, USA). One-way analysis of variance (ANOVA) was conducted using SPSS Statistics (version 27.0, IBM Corp., Armonk, NY, USA), followed by Tukey’s post hoc test for multiple comparisons. Differences were considered statistically significant at *p* < 0.05.

## 3. Results and Discussion

### 3.1. Effects of Different Starter Cultures on pH Value, Moisture Content and Color of Fermented Surimi Balls

Table 1 illustrates the changes in pH, moisture content, and color parameters of fermented large yellow croaker surimi balls inoculated with different starter cultures. Under weakly acidic conditions, fish myofibrillar proteins can form high-quality gels even without heating [35]. Moreover, a decrease in pH effectively inhibits the growth of spoilage and pathogenic microorganisms in fermented products, thereby enhancing their safety [36]. As shown in Table 1, both B and Y significantly reduced the pH of the surimi balls (*p* < 0.05), indicating substantial acid production during fermentation. The pH value in the Y group was lower than that in B, likely due to organic acids generated through the tricarboxylic acid (TCA) cycle during yeast metabolism [37].

Moisture is a key component of surimi balls, influencing their physical and rheological properties, and directly affecting textural characteristics and stability [38,39]. Compared with the CTR group, the moisture content in the B group decreased significantly (*p* < 0.05), which may be attributed to the contraction of the protein network and reduced water-holding capacity resulting from lactic acid bacteria fermentation [40]. In contrast, the moisture content in the Y group was slightly higher than that in CTR (*p* > 0.05), possibly due to the formation of hydrophilic colloids between extracellular polysaccharides secreted by yeast and the surimi protein matrix, thereby improving water retention [41]. The intermediate moisture content in the CTR group further confirms that fermentation influences the water-holding capacity of surimi balls.

The color of surimi balls considerably affects consumer appeal and purchasing decisions. As presented in Table 1, the type of starter culture had a significant impact (*p* < 0.05) on the L*, a*, b*, and whiteness (W) values of the fermented products. Compared with CTR, fermentation significantly decreased lightness (L*), increased redness (a* turned from negative to positive), and reduced whiteness (W). The slight darkening and reddening in the B group are mainly attributed to acid-induced partial denaturation of myoglobin by lactic acid bacteria [42], while the highest yellowness (b*) may be related to the accumulation of metabolites such as riboflavin [43]. The Y group exhibited the darkest and reddest color, along with the greatest loss in whiteness. In addition to myoglobin denaturation, these changes may be associated with Maillard reactions and pigment formation during yeast metabolism [44], as well as the formation of melanoidins via reactions between reducing sugars and amino compounds [45].

### 3.2. Effects of Different Starter Cultures on Texture Properties of Fermented Surimi Balls

Gel properties are critical indicators for evaluating the quality of surimi-based products, reflecting characteristics such as extensibility, elasticity, viscosity, and hardness. As shown in Table 2, except for springiness, all textural properties of fermented large yellow croaker surimi balls were significantly influenced by the type of starter culture used (*p* < 0.05).

In terms of hardness, the Y group exhibited a significant increase (57% higher than the CTR group), suggesting that yeast metabolism may enhance intermolecular cross-linking of myofibrillar proteins via the activation of transglutaminase pathways, thereby strengthening the gel structure [46,47]. Although the B group also showed a significant increase in hardness compared with CTR (28% higher), the lower magnitude suggests that acid-induced isoelectric aggregation of proteins by lactic acid bacteria contributes relatively less to gel strength [48,49].

A similar trend was observed for gumminess and chewiness, both of which were highest in the Y group. This further supports the role of yeast fermentation in promoting a denser and more chew-resistant gel matrix. However, resilience was significantly lower in the Y group than in the other groups, indicating a reduced capacity to recover after deformation. This may be attributed to the formation of micro-gas chambers within the protein network due to CO_2_ production during yeast fermentation, which could compromise network continuity and elasticity (as reflected in the slightly lower springiness of Y), potentially leading to a dough-like mouthfeel during prolonged chewing [50,51].

Regarding adhesiveness and structural stability, the absolute adhesiveness value of the B group (−8.49 g·s) was approximately 10 times higher than that of CTR, likely due to the viscous surface coating formed by exopolysaccharides produced through lactic acid bacterial metabolism [52]. In contrast, the Y group maintained low adhesiveness similar to CTR, indicating that yeast activity primarily modifies the internal gel structure without forming a highly viscous surface layer. No significant differences in springiness were observed among the groups (*p* > 0.05), suggesting that the 4-h fermentation period did not disrupt the fundamental actomyosin structure responsible for surimi ball elasticity. Cohesiveness was highest in CTR and lowest in B, indicating that acid production by lactic acid bacteria may induce partial dissociation and denaturation of proteins, weakening internal gel bonds [48]. The intermediate cohesiveness in the Y group implies a balance between moderate acidification and enzyme-mediated protein cross-linking [46].

Hence, under the fermentation conditions of 25 °C for 4 h, yeast fermentation significantly enhanced the hardness, gumminess, and chewiness of surimi balls. However, the accompanying decrease in resilience may require optimization of fermentation time or co-cultivation with lactic acid bacteria. Meanwhile, lactic acid bacteria fermentation moderately improved texture but resulted in excessively high adhesiveness and reduced cohesiveness.

### 3.3. Effects of Different Starter Cultures on Sensory Properties of Fermented Surimi Balls

**Figure 1:**
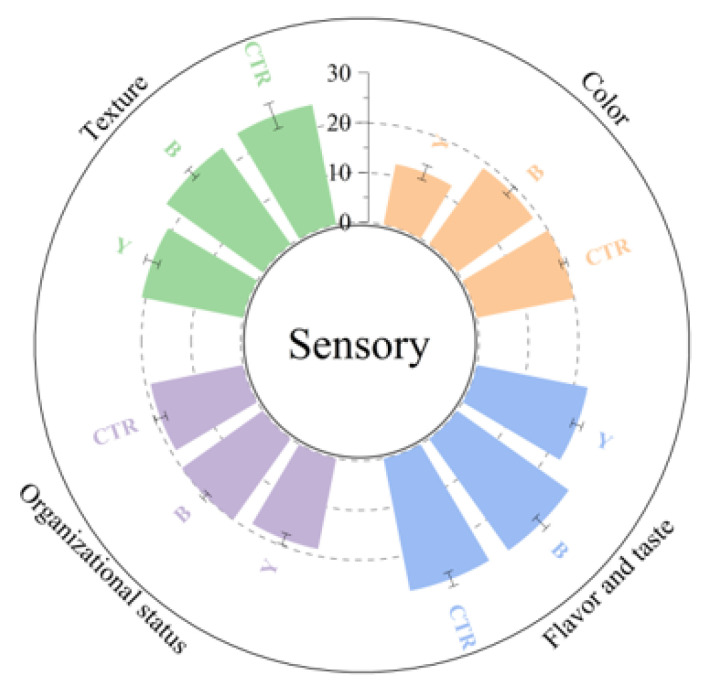
Effects of different starter cultures on the sensory properties of fermented large yellow croaker surimi balls. CTR = control (no starter culture), B = yogurt starter, and Y = dry yeast.

Sensory evaluation is a fundamental method for assessing and ensuring the quality of fish products, as it directly reflects consumer preferences [3]. According to the results from Figure 1 and the ANOVA analysis in Table A2, significant differences (*p* < 0.05) were observed among treatment groups in all sensory attributes except texture uniformity, indicating that the type of starter culture is a key factor influencing the sensory characteristics of fermented large yellow croaker surimi balls.

In terms of color, both CTR and B received significantly higher scores than Y (*p* < 0.05), while no significant difference was found between CTR and B. This suggests that the sterile system (CTR) effectively preserved the natural color of the surimi balls. The slight decrease in pH resulting from lactic acid bacteria (B) fermentation may have induced minor changes in protein structure, yet its impact on color remained limited. In contrast, yeast (Y) fermentation likely promoted the Maillard reaction and lipid oxidation, leading to pronounced browning [44,45]. Additionally, the inherent color of wheat bran further contributed to a substantial decrease in color acceptability.

For texture uniformity, no significant differences were detected among the groups (*p* > 0.05), indicating that short-term fermentation did not markedly alter the gel network structure of the surimi, regardless of microbial activity. It is worth noting that the B group received slightly higher scores, implying that lactic acid bacteria fermentation may mildly improve the uniformity of the gel structure, though not significantly (*p* > 0.05) [53].

Regarding texture, the CTR group scored significantly higher than Y (*p* < 0.05), while B showed no significant difference from either. This indicates that lactic acid bacteria fermentation had minimal adverse effects on texture, whereas yeast fermentation considerably modified protein cross-linking patterns [46,50], resulting in inferior texture properties. These results are consistent with the texture profile analysis, which showed that the Y group had the highest hardness and the lowest resilience.

In the category of flavor and taste, both CTR and B scored significantly higher than Y (*p* < 0.05), with no significant difference between them. This suggests that lactic acid bacteria fermentation may produce pleasant flavor-enhancing compounds, such as organic acids, acetoin, and diacetyl [48], contributing to a mildly improved flavor profile. On the other hand, yeast fermentation likely generated various volatile compounds, including alcohols and higher alcohols that are incompatible with the surimi matrix [54], along with inherent flavors from the wheat bran substrate. The combination of these off-flavors conflicted with the original umami and aromatic notes of the surimi, leading to significantly reduced flavor acceptance.

The total sensory scores showed no significant difference between CTR and B, both of which were significantly higher than that of Y (*p* < 0.05). Therefore, under the applied conditions of 25 °C and 4 h of fermentation, lactic acid bacteria (B) can serve as a favorable alternative to sterile fermentation, effectively maintaining the textural properties of surimi balls while optimizing their flavor. In contrast, yeast (Y) is presently unsuitable for single-strain fermentation of large yellow croaker surimi balls due to its negative effects on color, texture, and overall flavor.

### 3.4. Correlation Analysis of Physical and Chemical Indicators

**Figure 2:**
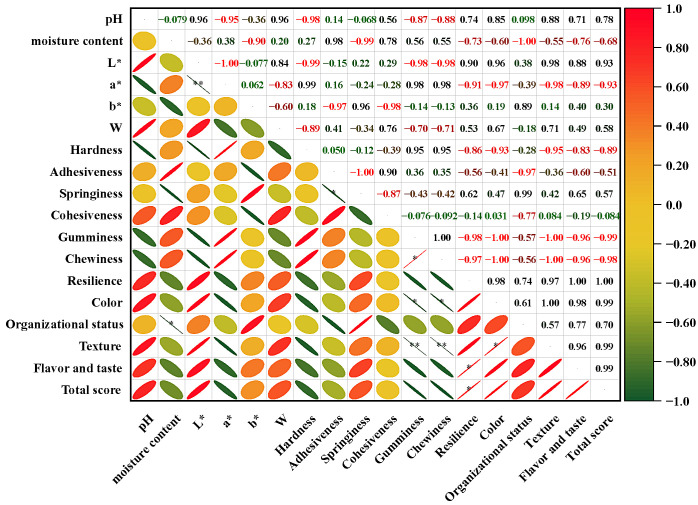
Correlation coefficients among physicochemical indicators of fermented large yellow croaker surimi balls. CTR = control (no starter culture), B = yogurt starter, and Y = dry yeast. The asterisk (*) represents significant difference. *: *p* < 0.05; **: *p* < 0.01.

To compare the physicochemical quality of fermented large yellow croaker surimi balls under different starter cultures and explore the interrelationships among various indicators, Pearson correlation analysis was conducted (Figure 2). The results revealed that pH served as a core factor driving quality changes. It showed highly significant positive correlations with lightness (L*), whiteness (W), and sensory scores for color and texture (r = 0.96, 0.96, 0.85, and 0.88, respectively), and a strongly significant negative correlation with redness (a*) (r = −0.95). This confirms that the pH decrease induced by fermentation is the primary cause of darkening and the increase in red values.

Analysis of textural properties indicated that hardness, gumminess, and chewiness were highly consistent (r = 0.95), collectively defining the texture network. However, these three parameters were significantly negatively correlated with resilience (r = −0.86 to −0.98), indicating that excessive hardness considerably reduces the elastic recovery capacity [50,51]. This explains why the Y group, despite its high hardness, received lower sensory acceptance in texture uniformity. The strong negative correlation between pH and hardness (r = −0.98) suggests that an acidic environment promotes myofibrillar protein cross-linking [47], thereby increasing the hardness of surimi balls.

Moisture content was almost perfectly negatively correlated with springiness and organizational status (r = −0.99 and −1.00, respectively), underscoring the critical role of water retention in maintaining structural integrity [55]. Moreover, a strong positive correlation was observed between moisture content and adhesiveness (r = 0.98), implying that exopolysaccharides produced by lactic acid bacteria form a highly adhesive surface layer, mitigating the negative effects of moisture reduction [56].

In terms of sensory attributes, the total sensory score was highly positively correlated with pH, L*, resilience, and flavor and taste (r = 0.78 to 1.00), indicating that bright color, elastic texture, and pleasant flavor are critical determinants of consumer acceptability. The significantly lowest total score of Y can be attributed to color deterioration, poor resilience, and the potential presence of incompatible flavor compounds. In contrast, B, benefiting from mild acidification and positive metabolic products, showed no significant difference in total score compared to CTR.

In summary, correlation analysis quantitatively confirmed that under the fermentation conditions of 25 °C for 4 h, B is a feasible alternative to sterile fermentation. Further optimization of inoculation amount and fermentation time may enhance product quality. On the other hand, metabolic pathway regulation is recommended for Y to alleviate its textural defects and flavor-related issues.

### 3.5. Effects of Different Starter Cultures on Free Amino Acid Contents of Fermented Surimi Balls

**Figure 3:**
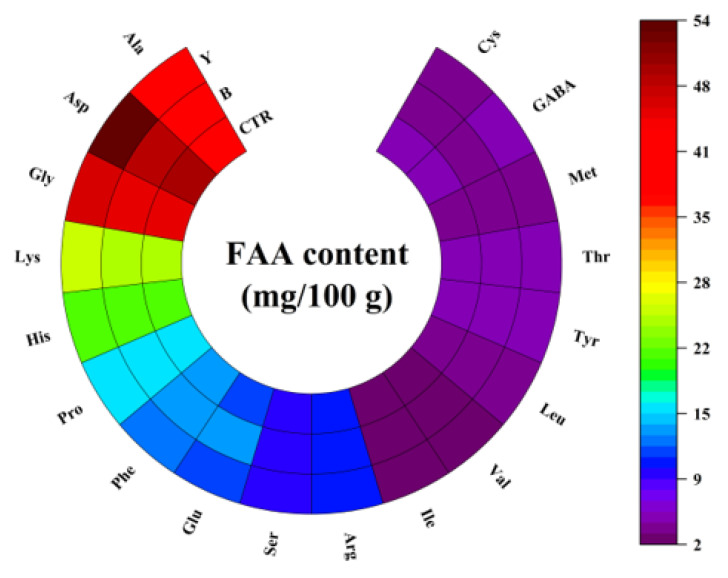
Effects of different starter cultures on the free amino acid composition of fermented large yellow croaker surimi balls. The heatmap color gradient represents differences in free amino acid contents, with purple indicating lower concentrations and red indicating higher concentrations. CTR, control without starter culture; B, inoculated with yogurt starter; Y, inoculated with dry yeast.

**Figure 4:**
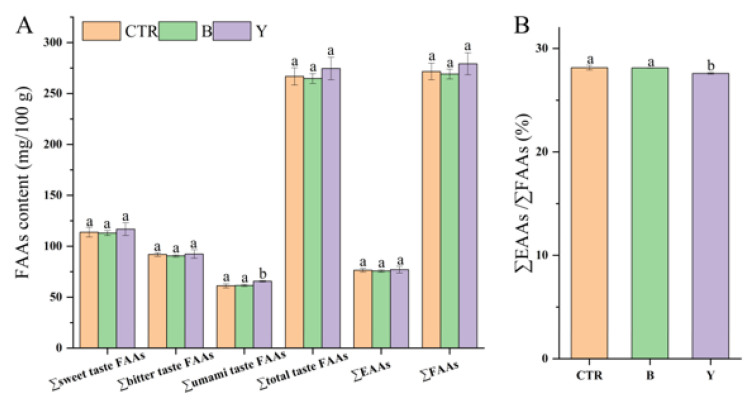
Free amino acid (FAA) contents (**A**) and the ratio of essential amino acids to total FAAs (**B**) in fermented large yellow croaker surimi balls inoculated with different starter cultures. Different letters above bars indicate significant differences among groups (*p* < 0.05). CTR denotes control (no starter culture), B denotes yogurt starter, and Y denotes dry yeast.

The flavor of aquatic products is strongly influenced by free amino acids (FAAs), as many act as direct taste contributors or as precursors of volatile compounds [29]. The composition and contents of FAAs in fermented large yellow croaker surimi balls inoculated with different starter cultures are shown in Figure 3 and Figure 4. Overall, the total FAA content (∑FAAs) showed no significant differences among groups, indicating that short-term fermentation had limited effect on overall protein hydrolysis. Similarly, the total content of bitter-taste FAAs (∑bitter taste FAAs) remained unchanged, suggesting that short-term fermentation did not cause undesirable bitterness. However, the FAA composition was significantly altered (*p* < 0.05) depending on the starter culture, reflecting strain-specific metabolic activities.

The most notable changes occurred in the Y group. Aspartate (Asp) content increased significantly to 53.88 mg/100 g, about 9.8% higher than CTR, contributing to a significant increase in the total umami FAAs (∑umami taste FAAs) to 65.50 mg/100 g, compared with 61.11 mg/100 g in CTR and 61.42 mg/100 g in B. This suggests that yeast may synergistically utilized acid proteases and glutaminases to selectively release Asp from protein substrates, thereby enhancing the umami perception [57,58]. Additionally, sweet-taste amino acids such as Gly, Ala, and Thr were slightly higher in Y compared with CTR and B (*p* > 0.05), which may help balance the sharp acidity of elevated Asp, producing a rounder flavor profile [59].

In contrast, B exhibited distinct metabolic characteristics, significantly reducing cysteine (Cys) and phenylalanine (Phe) contents, indicating strong utilization or conversion of sulfur-containing and aromatic amino acids. This reduced the accumulation of compounds associated with undesirable flavors while maintaining stable umami FAA levels, resulting in a more balanced flavor profile [60]. Furthermore, the ratio of essential amino acids to total FAAs (∑EAAs/∑FAAs) was significantly lowest in the Y group (*p* < 0.05, Figure 4B), implying that yeast may preferentially consumed essential amino acids as nitrogen sources, thereby slightly lowering the nutritional value.

Compared with CTR, B effectively reduced bitter-(Phe) and pungent (Cys) while maintaining umami FAAs, optimizing flavor balance. Y significantly enhanced umami intensity through Asp accumulation but at the expense of essential amino acids, making it more suitable for applications where strong umami is prioritized.

### 3.6. Effects of Different Starter Cultures on Flavor Nucleotides and Taste Activity Values of Fermented Surimi Balls

The umami taste of surimi balls is influenced not only by free amino acids but also by flavor nucleotides, which play a critical role in taste development [29]. Table 3 presents the effects of different starter cultures on the content of flavor nucleotides and their taste activity values (TAVs) in fermented large yellow croaker surimi balls. Generally, the type of starter culture significantly affected nucleotide accumulation and degradation (*p* < 0.05). For inosine monophosphate (IMP), the primary umami nucleotide, no significant differences were observed among the three groups (*p* > 0.05). The TAV of IMP was greater than 1 in all samples, reinforcing its role as a key contributor to umami taste in surimi balls and suggesting that fermentation did not substantially alter its content. In contrast, adenosine monophosphate (AMP) content varied significantly among groups, with the Y group showing the highest level. However, its TAV was substantially below 1, indicating only a minor direct contribution to umami intensity.

The equivalent umami concentration (EUC) was highest in the B group, suggesting that lactic acid bacteria fermentation enhanced umami synergy [61]. Furthermore, ATP and ADP contents were significantly lower in the Y group compared to CTR and B, while AMP and hypoxanthine (HX) were notably elevated. This indicates that yeast metabolism rapidly consumed ATP/ADP, thereby promoting AMP accumulation and accelerating the conversion of inosine (HXR) to HX. These results suggest that yeast fermentation enhanced nucleotide degradation, potentially increasing flavor complexity and imparting a stronger bitter note [54,62]. This observation is consistent with the FAA results, which showed the highest total FAAs and bitter-taste FAAs in the Y group.

In summary, lactic acid bacteria (B) fermentation preserved high IMP content and the highest EUC value, thereby strengthening the umami intensity of surimi balls. In contrast, yeast (Y) fermentation significantly shifted the nucleotide metabolic profile, particularly promoting AMP accumulation and HX formation, which may impart greater flavor complexity but also a more pronounced bitter undertone.

### 3.7. Effects of Different Starter Cultures on MDA, Carbonyl and Sulfhydryl Content of Fermented Surimi Balls

**Figure 5:**
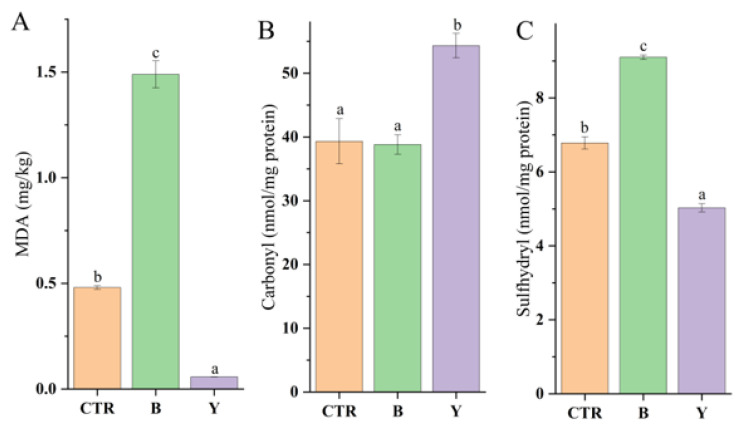
Effects of different starter cultures on the MDA (**A**), carbonyl (**B**) and sulfhydryl (**C**) content of fermented large yellow croaker surimi balls. Different letters in the same index indicate significant differences between groups (*p* < 0.05). CTR denotes the control (no starter culture), B denotes yogurt starter culture, and Y denotes dry yeast starter culture.

Figure 5 illustrates the effects of different starter cultures on the extent of protein oxidation and structural stability in fermented large yellow croaker surimi balls. As indicated, the type of starter culture significantly influenced the contents of malondialdehyde (MDA), carbonyl groups, and sulfhydryl groups (*p* < 0.05), demonstrating distinct microbial impacts on protein oxidative modification.

The lipid peroxidation indicator MDA was lowest in the Y group, significantly lower than in CTR and B, suggesting that yeast effectively suppressed the accumulation of lipid peroxidation end-products, likely due to the secretion of antioxidant compounds or reductive metabolites [63]. In contrast, the highest MDA content was observed in the B group, which may be attributed to hydrogen peroxide generated during lactic acid bacteria metabolism and the acidic environment that promoted secondary oxidation and facilitated MDA accumulation [64,65].

Protein oxidation markers showed distinct trends: carbonyl content was significantly higher in the Y group compared to CTR and B, indicating that acidic proteases from yeast not only enhanced proteolysis but also promoted carbonyl formation. Conversely, the sulfhydryl group content exhibited an opposite pattern, with the B group showing significantly higher levels than both CTR and Y. This suggests that the antioxidant system of lactic acid bacteria (e.g., the glutathione cycle) was activated early in fermentation, helping to protect sulfhydryl groups from oxidation and thereby supporting protein structural stability [65,66].

In summary, different starter cultures demonstrated contrasting oxidative behaviors during surimi ball fermentation. Y exhibited strong inhibition of lipid oxidation but facilitated protein oxidation, which may adversely affect gel texture. Although B led to increased lipid oxidation, its notably higher sulfhydryl content effectively suppressed the protein oxidation chain reaction [67], thereby preserving the integrity of the myofibrillar protein network.

### 3.8. Effects of Different Starter Cultures on Na^+^ and K^+^ Content of Fermented Surimi Balls

Inorganic ions significantly influence the sweetness and umami taste of seafood products and can enhance the release of flavor compounds from organic components [68]. As shown in Table 4, fermentation with different starter cultures significantly altered the Na^+^ and K^+^ concentrations in large yellow croaker surimi balls (*p* < 0.05), directly affecting the perceived saltiness intensity.

The Na^+^ content was markedly higher in both B and Y groups compared to CTR, and the taste activity value (TAV) was significantly greater than 1, indicating that Na^+^ is the primary ion contributing to salty taste. Fermentation likely facilitated the release or retention of Na^+^ through microbial metabolic activity [69], thereby enhancing salty perception. A similar trend was observed for K^+^, with TAV also exceeding 1, though its contribution to taste was considerably weaker than that of Na^+^. Both microbial starters, particularly B, significantly promoted the accumulation of K^+^, possibly due to ion release from microbial cell lysis or metabolic processes [69]. Overall, microbial fermentation, especially with lactic acid bacteria (B), significantly increased the Na^+^ and K^+^ contents in surimi balls without additional salt supplementation, thereby effectively intensifying salty taste. These results suggest that selected starter cultures can serve as a potential strategy for reducing added salt while improving umami perception in surimi products.

### 3.9. Correlation Analysis of Water-Soluble Indicators

**Figure 6:**
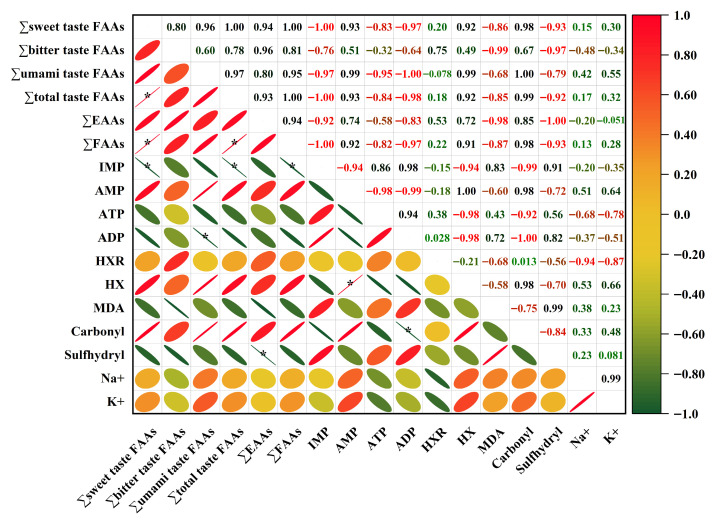
Correlation coefficients among water-soluble indicators of fermented large yellow croaker surimi balls. CTR denotes the control (no starter culture), B denotes yogurt starter culture, and Y denotes dry yeast starter culture. The asterisk (*) represents significant difference. *: *p* < 0.05.

To compare the effects of different starter cultures on flavor compounds, protein oxidation characteristics, and inorganic ion content in fermented large yellow croaker surimi balls, and to explore the intrinsic relationships among water-soluble components, a Pearson correlation analysis was performed (Figure 6). The results showed that the type of starter culture significantly influenced the comprehensive quality of surimi balls, with correlations categorized into four main pathways.

First, the umami-nucleotide pathway revealed a highly significant negative correlation between IMP and total umami free amino acids (∑umami taste FAAs) (r = −0.97), while IMP showed significant positive correlations with ADP (r = 0.98) and ATP (r = 0.86). This suggests that IMP accumulation was driven by rapid ATP/ADP consumption. Hypoxanthine (HX) was strongly positively correlated with AMP (r = 1.00) and total umami FAAs (r = 0.99), supporting the notion that yeast fermentation drives the AMP → IMP → HXR → HX cascade [70], thereby enhancing flavor complexity and bitterness.

Second, within the sweet-bitter amino acid pathway, total sweet-taste FAAs were significantly positively correlated with total umami FAAs (r = 0.96), and both were highly consistent with total FAAs (r = 1.00 and 0.95, respectively), indicating that concurrent proteolysis is the common source for the increase in both types of amino acids.

Third, the oxidative stress pathway showed negative correlations between carbonyl content and MDA (r = −0.75) and sulfhydryl groups (r = −0.84), whereas sulfhydryl content was positively correlated with MDA (r = 0.99). This implies that sulfhydryl depletion is a key node in the synergistic oxidation of lipids and proteins [71]. The strong negative correlation between sulfhydryl content and total essential amino acids (r = −1.00) suggests that protein oxidation contributes to the loss of essential amino acids. The high sulfhydryl levels in the B group effectively suppressed oxidative chain reactions [67].

Finally, in the inorganic ion-taste pathway, Na^+^ and K^+^ were strongly positively correlated (r = 0.99). However, both ions were negatively correlated with IMP and ATP, and positively correlated with HX and carbonyl content. This indicates that ion accumulation is accompanied by nucleotide degradation and protein oxidation, collectively contributing to a multifaceted flavor profile combining salty, bitter, and umami notes [72,73].

In summary, lactic acid bacteria and yeast fermentation each offer distinct advantages in modifying the flavor of surimi balls. B promoted a balanced taste and improved protein structural stability, whereas Y enhanced nucleotide and protein degradation, resulting in a more complex flavor profile.

## 4. Conclusions

This study demonstrates that direct vat set (DVS) starters provide a viable and efficient strategy for tailoring fermented large yellow croaker surimi quality. Lactic acid bacterial fermentation simultaneously enhances protein stability, maintains a balanced umami profile and preserves gel resilience, making it the preferred route for industrial product development. Yeast fermentation, while intensifying hardness and umami intensity, compromises color and protein oxidative stability; these drawbacks necessitate further optimization (e.g., shorter fermentation or mixed cultures). Overall, the findings establish a theoretical basis for selectively deploying commercial non-aquatic starters in aquatic value-added processing. Future work should refine fermentation parameters and exploit co-culture systems to synergize the complementary metabolic advantages of different microbial strains.

## Figures and Tables

**Table 1:** Effects of different starter cultures on the pH value, moisture content and color of fermented large yellow croaker surimi balls.

Sample	pH	Moisture Content (%)	Color			
			L*	a*	b*	W
CTR	7.26 ± 0.06 ^c^	70.91 ± 0.11 ^b^	84.72 ± 1.26 ^b^	−0.59 ± 0.07 ^a^	8.17 ± 0.71 ^a^	82.63 ± 0.94 ^b^
B	6.93 ± 0.03 ^b^	69.41 ± 0.46 ^a^	83.45 ± 2.52 ^ab^	0.36 ± 0.04 ^b^	11.70 ± 1.05 ^b^	79.66 ± 2.11 ^a^
Y	6.76 ± 0.04 ^a^	71.46 ± 0.11 ^b^	81.59 ± 1.95 ^a^	1.83 ± 0.14 ^c^	8.86 ± 0.72 ^a^	79.45 ± 1.76 ^a^

Values are mean ± SD. Different superscript letters within the same column indicate significant differences among groups (*p* < 0.05). CTR represents the control group (no starter culture), B denotes yogurt starter, and Y denotes dry yeast.

**Table 2:** Effects of different starter cultures on the texture properties of fermented large yellow croaker surimi balls.

Texture Index	CTR	B	Y
Hardness (g)	1748.99 ± 15.23 ^a^	2238.74 ± 144.97 ^b^	2741.85 ± 52.05 ^c^
Adhesiveness (g·s)	−0.800 ± 0.024 ^b^	−8.49 ± 0.770 ^a^	−0.415 ± 0.036 ^b^
Springiness	0.883 ± 0.020 ^a^	0.904 ± 0.030 ^a^	0.880 ± 0.020 ^a^
Cohesiveness	0.757 ± 0.017 ^b^	0.634 ± 0.056 ^a^	0.708 ± 0.036 ^ab^
Gumminess	1324.33 ± 35.86 ^a^	1449.48 ± 101.54 ^a^	1942.22 ± 127.46 ^b^
Chewiness	1162.52 ± 40.48 ^a^	1305.46 ± 73.18 ^a^	1818.14 ± 176.31 ^b^
Resilience	0.438 ± 0.016 ^b^	0.440 ± 0.023 ^b^	0.374 ± 0.024 ^a^

Values are mean ± SD. Different superscript letters within the same row indicate significant differences among groups (*p* < 0.05). CTR represents the control without starter culture, B indicates yogurt starter addition, and Y denotes dry yeast addition.

**Table 3:** Effects of different starter cultures on the flavor nucleotides and taste activity values of fermented large yellow croaker surimi balls.

Sample	IMP		AMP		EUC (%)	Content (mg/100 g)			
	Content (mg/100 g)	TAV	Content (mg/100 g)	TAV		ATP	ADP	HXR	HX
CTR	78.44 ± 1.81 ^a^	3.14	0.181 ± 0.011 ^a^	0.004	1.53	2.80 ± 0.34 ^b^	11.98 ± 1.65 ^b^	60.49 ± 5.56 ^b^	9.59 ± 0.91 ^a^
B	78.95 ± 4.19 ^a^	3.16	0.285 ± 0.003 ^b^	0.006	1.66	2.42 ± 0.13 ^b^	11.84 ± 0.63 ^b^	40.44 ± 1.50 ^a^	12.47 ± 0.72 ^b^
Y	76.08 ± 5.05 ^a^	3.04	0.760 ± 0.021 ^c^	0.015	1.47	1.77 ± 0.22 ^a^	2.28 ± 0.07 ^a^	50.20 ± 4.39 ^ab^	23.88 ± 4.39 ^c^

Values are mean ± SD. Different letters within the same column indicate significant differences among groups (*p* < 0.05). CTR denotes the control (no starter culture), B denotes yogurt starter culture, and Y denotes dry yeast starter culture.

**Table 4:** Effects of different starter cultures on the Na^+^ and K^+^ content of fermented large yellow croaker surimi balls.

Sample	Na+		K+	
	Content (mg/100 g)	TAV	Content (mg/100 g)	TAV
CTR	674.33 ± 6.13 ^a^	75.34	43.72 ± 2.34 ^a^	3.16
B	886.30 ± 5.26 ^c^	99.03	107.35 ± 9.40 ^b^	7.75
Y	851.26 ± 13.12 ^b^	95.11	107.34 ± 7.00 ^b^	7.75

Values are mean ± SD. Different letters within the same column indicate significant differences among groups (*p* < 0.05). CTR denotes the control (no starter culture), B denotes yogurt starter culture, and Y denotes dry yeast starter culture.

## Data Availability

The original contributions presented in the study are included in the article. Further inquiries can be directed to the corresponding authors.

## Appendix A

### Appendix A.1

**Table A1:** Standards for sensory evaluation.

Index	Evaluation Standards	Score
Color	light white, glossy	15~20
	gray white or slightly yellowish, with a slight luster	8~14
	light yellow, slightly glossy or dull	1~7
Organizational status	smooth surface, compact cut surface, no or minimal pores	15~20
	relatively smooth surface, basically compact cut surface, a few small pores	8~14
	surface roughness, loose cutting surface, numerous pores or dense pores	1~7
Texture	good elasticity and chewiness	21~30
	moderate elasticity, moderate chewiness	11~20
	poor or no elasticity, poor chewiness	1~10
Flavor and taste	rich or moderate fermentation flavor, distinct fresh taste of fish, no fishy smell, harmonious aroma, delicious	21~30
	plain fermentation flavor, plain fresh taste of fish, light fishy smell, average taste	11~20
	slightly sour or absent fermentation flavor, no fresh taste of fish, obvious fishy smell, poor taste	1~10

### Appendix A.2

**Table A2:** Effects of different starter cultures on the sensory properties of fermented large yellow croaker surimi balls.

Index	CTR	B	Y
Color	20.03 ± 0.71 ^b^	18.80 ± 1.10 ^b^	12.41 ± 1.52 ^a^
Organizational status	19.02 ± 1.22 ^a^	20.07 ± 0.71 ^a^	18.61 ± 1.14 ^a^
Texture	24.60 ± 2.70 ^b^	23.83 ± 1.10 ^ab^	20.80 ± 1.48 ^a^
Flavor and taste	27.22 ± 1.48 ^b^	27.61 ± 1.52 ^b^	22.84 ± 1.10 ^a^
Total score	90.88 ± 6.12 ^b^	90.32 ± 4.41 ^b^	74.66 ± 5.24 ^a^

Different letters within the same column indicate significant differences among groups (*p* < 0.05). CTR denotes the control (no starter culture), B denotes yogurt starter culture, and Y denotes dry yeast starter culture.
